# Trastuzumab Deruxtecan for brain metastatic squamous lung carcinoma with immune-related hypophysitis: a case report

**DOI:** 10.3389/fphar.2025.1608589

**Published:** 2025-09-11

**Authors:** Rui Xu, Jiadi Gan, Jiarui Zhang, Linhui Yang, Yi Liu, Weimin Li, Kaige Wang, Dan Liu

**Affiliations:** Department of Pulmonary and Critical Care Medicine, Institute of Respiratory Health, Center of Precision Medicine, West China Hospital, Sichuan University, Chengdu, Sichuan, China

**Keywords:** antibody-drug conjugates, targeted therapy, immunotherapy, immune-related hypophysitis, immune-related adverse events

## Abstract

Immunotherapy is an important part of the first-line treatment for lung squamous cell carcinoma (LUSC) in the current guidelines. However, in case when patients experience immunotherapy-related adverse reactions, immunotherapy may be discontinued. Antibody-drug conjugates (ADCs) may be an alternative treatment if the patient harbors actionable mutations. Herein, we present a case of brain metastatic LUSC harboring *HER2* mutation who experienced hypophysitis after immunotherapy. This patient benefited from Trastuzumab Deruxtecan (T-DXd) after discontinuing immunotherapy. Therefore, T-DXd may be an alternative treatment option for LUSC patients with *HER2* mutation who are unable to continue immunotherapy.

## Introduction

Non-small-cell lung cancer (NSCLC) is cancer with the highest morbidity and mortality, of which 20%–30% are the squamous subtype (Lau et al., 2022). Immunotherapy combined with chemotherapy is suggested as one of the first-line treatments for advanced lung squamous cell carcinoma (LUSC) (Cheng et al., 2021). Within all immunotherapy-related adverse events (irAEs), immune-related hypophysitis (irH) is a relatively rare occurrence (<1%). However, severe irAEs may present a barrier to continued immunotherapy (Remon et al., 2018). The emerging targeted therapies based on driver genes like antibody-drug conjugates (ADCs), may offer a potential reversal of this predicament.

Human epidermal growth factor receptor-2 (*HER2*) is one of the driver genes in NSCLC. The incidence of *HER2* mutation, amplification, and overexpression is 2%–4%, 10%–20%, and 6%–35%, respectively (Mar et al., 2015). Furthermore, approximately 47% of patients with *HER2*-mutated NSCLC are diagnosed with brain metastases at initial diagnosis or during treatment (Offin et al., 2019). Overall, intracellular signaling pathways induced by the *HER2* promote processes that are favorable to tumor progression in LUSC, similar to adenocarcinoma (Loeffler et al., 2023). Trastuzumab Deruxtecan (T-DXd), targeting *HER2*, is the world’s first ADCs in lung cancer, lately presenting prominent outcomes with a median progression-free survival (PFS) of 9.9 months and median overall survival (OS) of 19.5 months in the 5.4 mg/kg group of DESTINY-Lung02 (Goto et al., 2023). A comprehensive pooled analysis further reveals a PFS of 7.1 months and an OS of 13.6 months in *HER2*-mutated non-small cell lung cancer patients with brain metastases (Li et al., 2023). Despite its marked efficacy, it’s noteworthy that only one participant with a histological classification of squamous cancer was included in prior trials (Goto et al., 2023; Li et al., 2022). Since *HER2* mutations are relatively rare in LUSC, real-world evidence validating the efficacy of T-DXd in LUSC patients remains conspicuously absent.

## Case report

A 69-year-old male with no significant past medical history or comorbidities was found to have a pulmonary mass in the left lung during a routine physical examination. The patient is a never smoker, retired office worker and married. He reported no known exposure to carcinogens (asbestos, silica, or heavy metals) and his family history was negative for malignancies in first-degree relatives. Left upper lobectomy with lymph node dissection was subsequently performed and postoperative pathology confirmed a stage 3B (pT3N2M0) LUSC. The 1021-gene next-generation sequencing (NGS) panel (platform: illumina, coverage: exceeding 500×) identified a *HER2* exon 20 insertion p.772_775dup and a *KRAS* p.G12S mutation, with *EGFR*, *ALK*, *ROS-1*, *BRAF*, *MET* mutations negative. Additionally, programmed death-ligand 1 (PD-L1) expression was assessed by immunohistochemistry (IHC) and found to be 60%. In the 3-month post-operation follow-up, cranial magnetic resonance imaging (MRI) revealed multiple intracranial metastases. Consequently, the patient commenced first-line therapy with pembrolizumab, combined with carboplatin and nab-paclitaxel chemotherapy, alongside concurrent whole-brain radiotherapy. The cranial MRI after 3 months of first-line therapy demonstrated a reduction in the size of the intracranial lesion. The patient subsequently received pembrolizumab as maintenance therapy, in combination with three additional courses of cranial radiotherapy.

**FIGURE 1:**
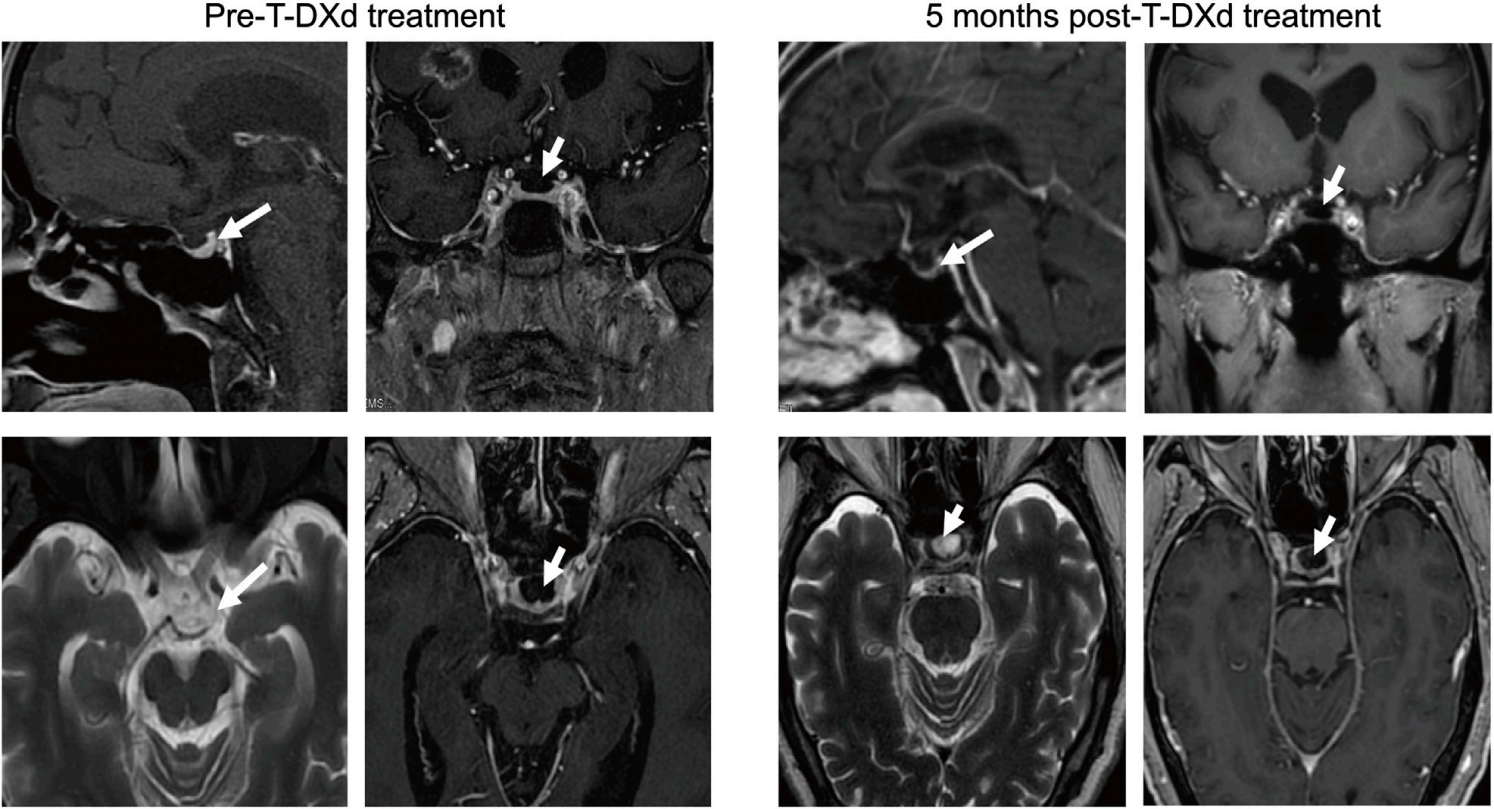
The patient’s cranial magnetic resonance imaging showed no improvement in empty sella turcica after Trastuzumab Deruxtecan treatment. The pituitary gland volume is reduced to a concave shape, and the empty sella turcica is present (white arrows).

After 22 months of pembrolizumab maintenance therapy, the patient reported symptoms of dizziness and fatigue. The adrenal function test was performed and showed low levels of adrenocorticotropic hormone (ACTH) (1.30 ng/L, normally range 5.00–78.00 ng/L) and corticosteroid hormone (47.60 nmol/L, normally range 33.00–537.00 nmol/L). The cranial MRI indicated a progression of the cranial lesion and presence of an empty sella turcica (Figure 1). The patient was diagnosed with irH and secondary adrenal insufficiency, which was attributed to an adverse reaction to immune checkpoint inhibitors (ICIs). Glucocorticoid (GC) was administered and his symptoms were significantly relieved.

**FIGURE 2:**
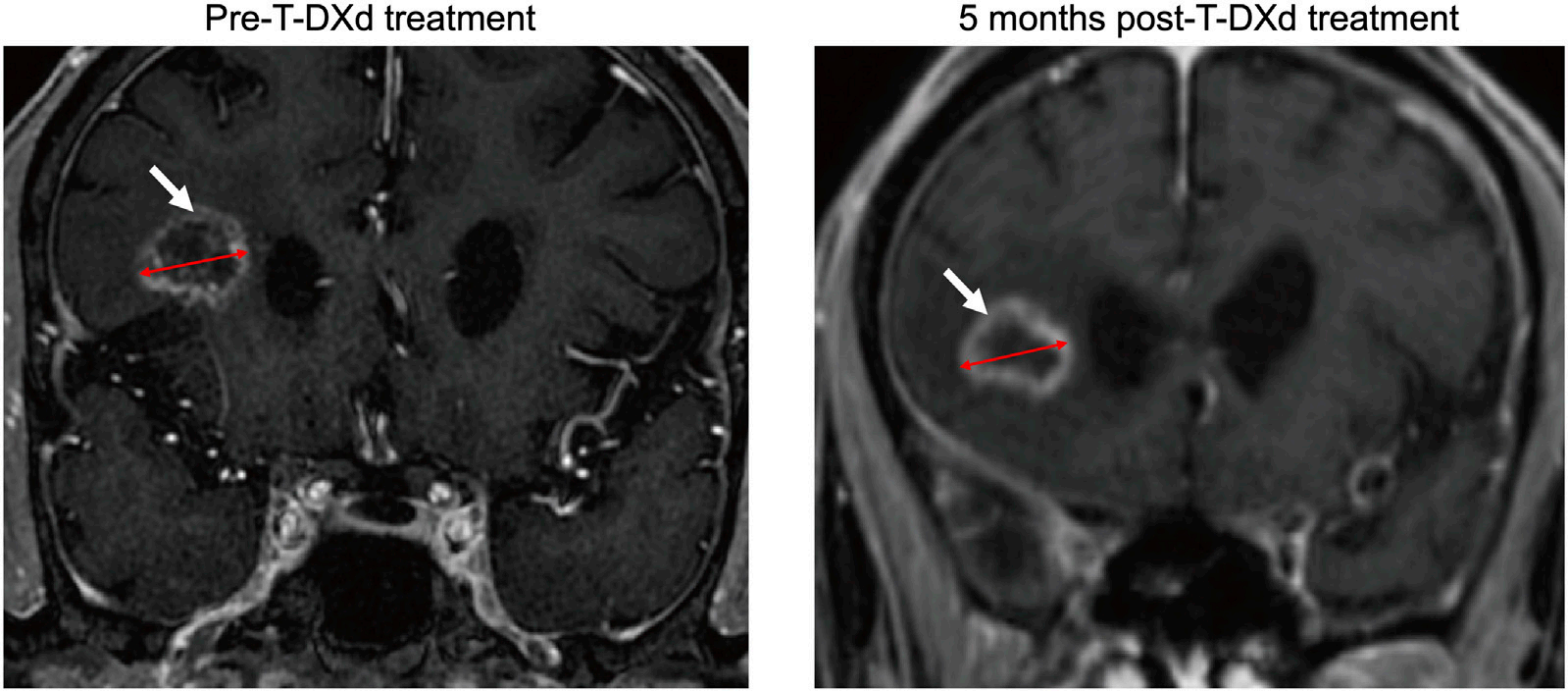
Magnetic resonance imaging of brain metastasis (white arrows) before and after five months of Trastuzumab Deruxtecan treatment. The red double-headed arrows indicate the longest diameter of the lesion.

However, the patient could no longer tolerate immunotherapy in this condition. Considering this patient harbored a *HER2* mutation, ADCs such as T-DXd were considered as an optional anti-tumor treatment in this condition. Following informed consent, the patient received T-DXd (300 mg q3w) as a subsequent anti-tumor therapy. Notably, an adverse event of nausea and retching was reported. After 1 month of T-DXd treatment, plasma ACTH and corticosteroid levels increased to 28.50 ng/L and 164.00 nmol/L, respectively, both within normal ranges. At 2 months, further increases were observed, with ACTH rising to 36.21 ng/L and cortisol to 192.00 nmol/L. This suggests that the patient’s secondary adrenal insufficiency has improved (Table 1). The patient received regular follow-up examinations at 6-week interval and were assessed as SD. The cranial MRI at the fifth month post-T-DXd treatment showed a maximum lesion diameter of 2.09 cm compared to the pre-T-DXd treatment measurement of 1.89 cm (Figure 2), consistent with stable disease (SD) according to the Response Evaluation Criteria in Solid Tumors version 1.1 (RECIST v1.1) and the Response Assessment in Neuro-Oncology (RANO) criteria. However, cranial MRI still indicated an empty sella turcica (Figure 1) while the patient’s ACTH level normalized. The patient reported relief of the dizziness and fatigue and tolerance of T-DXd treatment.

**TABLE 1:** Improvement in plasma ACTH and corticosteroid hormone levels throughout treatment with T-DXd.

	Plasma ACTH (ng/L)	Plasma corticosteroid (nmol/L)
Before T-DXd	1.30	47.60
1 month post-T-DXd	28.50	164.00
2 months post-T-DXd	36.21	192.00
Reference value	5.00–78.00	133.00–537.00

ACTH, Adrenocorticotropic hormone. T-DXd, Trastuzumab Deruxtecan.

**FIGURE 3:**
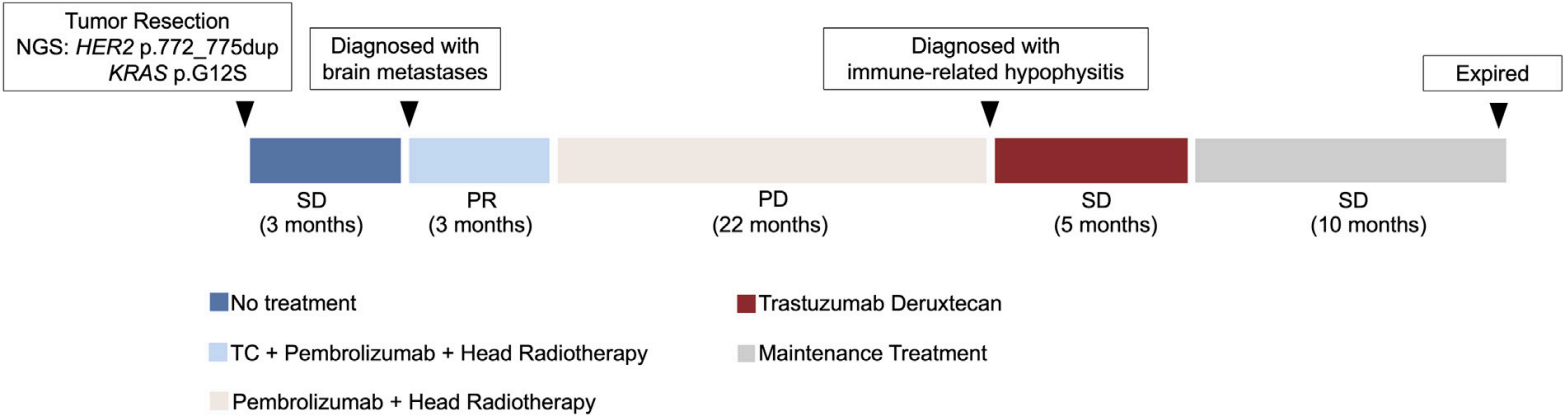
Timeline of the clinical diagnosis and treatment course. NGS, next-generation sequencing. TC, albumin-bound paclitaxel/carboplatin chemotherapy. T-DXd, Trastuzumab Deruxtecan.

At the sixth month of T-DXd treatment, the patient refused to continue T-DXd treatment. Thereafter, the patient did not receive any antitumour therapy. At the seventh month after cessation of T-DXd treatment, the patient underwent a final follow-up at a local hospital. It was reported that the intracranial lesion was stable without progression, and there was no recurrence in the lungs and no metastases in the remaining organs. According to the latest telephone follow-up, the patient died at the 10th month after cessation of T-DXd treatment. The course of this patient’s diagnosis and treatment is shown in Figure 3.

## Discussion

The occurrence of irH was less frequent with anti-PD-1 or PD-L1 treatment than with anti-CTLA-4 treatment: 0.4% with PD-1 inhibitors, 0.1% with PD-L1 vs. 3.4% with anti-CTLA-4 (Tan et al., 2019). Previous studies indicated that irH induced by anti-CTLA-4 implicates a type II hypersensitivity reaction with the activation of the classical complement pathway and secondary development of hypophysitis. The activation of this pathway could be explained by an ‘ectopic’ pituitary expression of CTLA-4 antigens on prolactin in mice model (Iwama et al., 2014; Quirk et al., 2015). As for irH induced by anti-PD-1 such as pembrolizumab, the pathogenesis of the few anti-PD-1 or PD-L1-induced cases of hypophysitis is not clear as IgG4 cannot activate the complement pathway and is less effective for antibody-dependent cell-mediated cytotoxicity (Vidarsson et al., 2014; Bruhns et al., 2009). It has the possibility to share a similar mechanism as IgG4-related hypophysitis as these antibodies are IgG4 (Bellastella et al., 2016), or PD-1 could be expressed in pituitary cells or lymphocytes (Mei et al., 2016). In this case, this patient showed normalized hormone levels after a recommended GC treatment but without a radiology improvement. The normalized hormone levels were attributed to the control of inflammation reactions and the recovery of hypothalamic pituitary adrenal axis (Khawaja et al., 2023). Regarding the non-improving radiology findings, we suppose it’s because empty sella turcica is the final stage of irH (Nakasone et al., 2015; Karaca et al., 2009), possibly due to the irreversible fibrosis of the hypophysitis tissue (Kurokawa et al., 2020).

In this case, the patient’s hormone returned to a normal level after a recommended GC treatment but imaging presentations generally do not improve even, as observed in this case (Albarel et al., 2019). ICIs re-dosing treatment remains controversial, attributed to a lack of existing data. ADCs are widely distributed in multiple solid tumor fields nowadays. Targets in lung cancer mainly include *HER2*, *HER3*, *TROP2*, *MET*, *CEACAM5*, *B7-H3* etc (Passaro et al., 2023). To the best of our knowledge, of T-DXd treatment for brain metastatic NSCLC cases that have been reported, all were lung adenocarcinomas. Huang et al. reported a case who received T-DXd treatment as first-line therapy and was observed with SD in brain metastases, achieving an OS of more than 1 year (Huang et al., 2025); Xu et al. reported an advanced lung adenocarcinoma case receiving T-DXd treatment after multiple lines therapy, and he achieved an OS of 46.5 months (Xu et al., 2024); Güren et al. reported a case that achieved complete remission after four cycles of T-DXd (Güren et al., 2024); Young et al. reported a case achieving an OS of more than 16 months who received T-DXd as a second-line therapy (Young et al., 2025). As for LUSC, it’s interesting that Xia et al. reported a case of a LUSC patient who harbored a *HER2* mutation presenting with progressive disease after immunotherapy, which may suggest reduced efficacy of immunotherapy in LUSC patients with *HER2* mutations. The undesirable response to ICIs in LUSC patients may attribute to the immune-excluded phenotypes in the LUSC immune microenvironment, which is not HER2-specific (Xia et al., 2021). In this case, this LUSC patient was accompanied by brain metastases and immunotherapy intolerant, which narrowed the subsequent treatment options. It’s known that TKIs targeting HER2 mutations (afatinib, dacomitinib, and pyrotinib, etc.) did not achieve an ORR of 30%, with a median PFS of 3–6.9 months, which is inferior to T-DXd. Moreover, since the predominant issue in this case was brain metastasis, we expected that TKIs would provide a limited benefit. Therefore, the patient and his family chose T-DXd as the subsequent treatment after being adequately informed.

In this case, we presented a case of a *HER2* mutation brain-metastatic LUSC patient who suffered from irH, received T-DXd treatment as next-line antitumor therapy and the tumor progression was under control. When LUSC patients are unable to continue immunotherapy as recommended by current guidelines, subsequent treatment should take into account the patient’s genetic test results and general condition. Currently, there are few specialized studies on T-DXd for the treatment of LUSC, and we aim to present a case of real-world application of T-DXd in the treatment of a relatively rare pathological type of lung cancer, aiming to provide some reference for clinical treatment. However, this study has its limitations. No biopsy was performed to obtain histologic confirmation of the recurrence or irH due to the patient’s advanced age and poor condition. Also, he received only 5 months of T-DXd treatment, and thus, the long-term safety of T-DXd is lacking. The information on the last follow-up was verbalized by the patient’s family and objective assessment parameters were missing. Nowadays, adenocarcinomas have more robust evidence of significant survival gains with HER2-targeted therapy, but there is still a vacancy in LUSC. Therefore, we suppose that future studies with larger sample sizes are required to explore the potential population benefiting from T-DXd treatment for LUSC patients with *HER2* mutation.

## Conclusion

T-DXd has the potential to expand treatment options for *HER2*-mutation LUSC, especially in patients who are unable to continue immunotherapy due to irAEs.

## Data Availability

The original contributions presented in the study are included in the article/supplementary material, further inquiries can be directed to the corresponding authors.

## Abbreviations

ACTH, adrenocorticotropic hormone; ADCs, antibody-drug conjugates; GC, glucocorticoid; *HER2*, human epidermal growth factor receptor-2; ICIs, immune checkpoint inhibitors; irAEs, immunotherapy-related adverse events; irH, immune-related hypophysitis; LUSC, lung squamous cell carcinoma; MRI, Magnetic Resonance Imaging; NGS, next-generation sequencing; NSCLC, non-small-cell lung cancer; OS, overall survival; PFS, progression-free survival; SD, stable disease; T-DXd, Trastuzumab Deruxtecan.
